# Supplementary material for: Inflammatory factors and risk of lung adenocarcinoma: a Mendelian randomization study mediated by blood metabolites
Source: Front Endocrinol (Lausanne). 2024 Aug 27;15:1446863. doi: 10.3389/fendo.2024.1446863 (PMC11384989; doi:10.3389/fendo.2024.1446863)
Supplement: Supplementary file 1 [file DataSheet1.zip › supplementary files/S2/GCST90200384/forest.pdf]

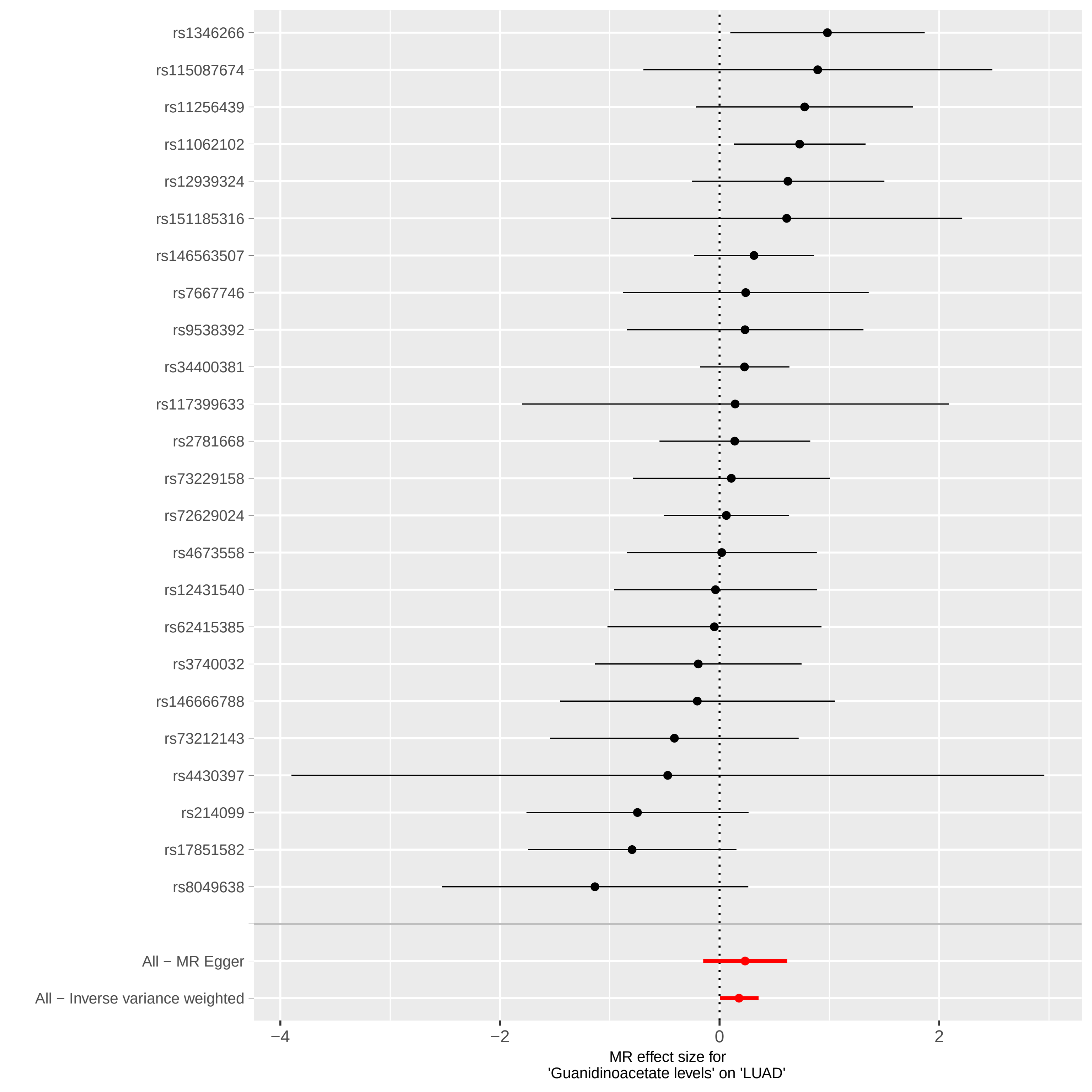

rs1346266
rs115087674
rs11256439
rs11062102
rs12939324
rs151185316
rs146563507
rs7667746
rs9538392
rs34400381
rs117399633
rs2781668
rs73229158
rs72629024
rs4673558
rs12431540
rs62415385
rs3740032
rs146666788
rs73212143
rs4430397
rs214099
rs17851582
rs8049638
All − MR Egger
All − Inverse variance weighted
−4
−2
0
2
MR effect size for
'Guanidinoacetate levels' on 'LUAD'
